# Protective Effects of Nintedanib against Polyhexamethylene Guanidine Phosphate-Induced Lung Fibrosis in Mice

**DOI:** 10.3390/molecules23081974

**Published:** 2018-08-07

**Authors:** Hyeon-Young Kim, Min-Seok Kim, Sung-Hwan Kim, Doin Joen, Kyuhong Lee

**Affiliations:** 1 National Center for Efficacy Evaluation of Respiratory Disease Product, Korea Institute of Toxicology, 30, Baekhak 1-gil, Jeongeup-si 56212, Korea; hyeonyoung.kim@kitox.re.kr (H.-Y.K.); sunghwan.kim@kitox.re.kr (S.-H.K.); doin.jeon@kitox.re.kr (D.J.); 2 Department of Toxicology Evaluation, Graduate School of Pre-Clinical Laboratory Science, Konyang University, Daejeon 35365, Korea; 3 Laboratory Animal Center, Daegu-Gyeongbuk Medical Innovation Foundation, Daegu 360-4, Korea; minseok.kim@kitox.re.kr; 4 Department of Human and Environmental Toxicology, University of Science & Technology, Daejeon 34113, Korea

**Keywords:** nintedanib, polyhexamethylene guanidine phosphate, pulmonary fibrosis, anti-fibrosis, inflammasome

## Abstract

Nintedanib (NDN), a tyrosine kinase inhibitor, has been shown to have anti-tumor, anti-inflammatory, and anti-fibrotic effects in several reports. We investigated the protective effects of NDN against polyhexamethylene guanidine phosphate (PHMG)-induced lung fibrosis in mice. The following three experimental groups were evaluated: (1) vehicle control; (2) PHMG (1.1 mg/kg); and (3) PHMG & NDN (60 mg/kg). PHMG induced pulmonary inflammation and fibrosis by intratracheal instillation in mice. In contrast, NDN treatment effectively alleviated the PHMG induced lung injury, and attenuated the number of total cells and inflammatory cells in the bronchoalveolar lavage fluid, including the fibrotic histopathological changes, and also reduced the hydroxyproline content. NDN also significantly decreased the expression of inflammatory cytokines and fibrotic factors, and the activation of the NLRP3 inflammasome in lung tissues. These results suggest that NDN may mitigate the inflammatory response and development of pulmonary fibrosis in the lungs of mice treated with PHMG.

## 1. Introduction

Idiopathic pulmonary fibrosis (IPF) is a severely debilitating and fatal lung disease, with median survival ranging from two to three years after diagnosis [1,2,3]. It is characterized by lung damage with a loss of alveolar epithelial cells and abnormal tissue repair, accumulation of abnormal fibroblasts and myofibroblasts, deposition of extracellular matrix, and distortion of pulmonary architecture, which result in respiratory failure [4,5]. The etiology of IPF is by definition unknown, although potential risk factors such as exposure to environmental sources have been described [6].

Polyhexamethylene guanidine phosphate (PHMG) is a water-soluble chemical related to the polymeric guanidine family [7]. In 2011, the Korea Centers for Disease Control and Prevention reported that fatal pulmonary disease may be caused by household humidifier disinfectants. The main ingredient of humidifier disinfectant products is PHMG, which prevents the growth of germs [8]. Inhalation of PHMG causes severe pulmonary injury and respiratory failure, characterized by the loss of parenchymal architecture and subsequent massive lung fibrosis [9]. The use of PHMG has been banned, yet many people suffer from its pulmonary effects. Additionally, the mechanism by which PHMG causes pulmonary fibrosis is not fully understood.

Nintedanib (NDN), previously known by its research code BIBF 1120, is an intracellular inhibitor of tyrosine kinases (TKs) approved by the US Food and Drug Administration (FDA), and was developed for the treatment of a number of cancer types such as those of the lung, kidney, and liver [10,11]. Recently, NDN has been found to have beneficial therapeutic effects on pulmonary fibrosis, as well as effective antitumor activity, and is being used in the treatment of IPF [12,13]. The results of two replicate phase III INPULSIS trials demonstrated that NDN reduced disease progression in IPF patients by improving respiratory function [14]. NDN is currently used for the treatment of patients whose lungs have been injured by PHMG, but its ability to improve PHMG-induced pulmonary fibrosis has not yet been studied.

The nucleotide-binding oligomerization domain (NOD)-like receptors family includes the pyrin domain-containing 3 (NLRP3; also known as the NALP3 or cryopyrin) inflammasome, which is a large multiprotein complex that plays a key role in innate immunity [15]. The NLRP3 inflammasome is a key factor that drives the progression of pulmonary fibrosis [16]. Our previous study showed that the administration of oleanolic acid acetate (OAA) inhibited the activation of the NLRP3 inflammasome and the development of pulmonary fibrosis in PHMG-induced lung injury in mice [17]. The activation of the NLRP3 inflammasome is involved in the regulation of inflammatory cytokine expression [18]. Cytokines are key modulators that induce an inflammatory response, and these responses are considered to play a crucial role in the pathogenesis of PHMG-induced pulmonary fibrosis [19,20,21].

In the present study, we investigated the effects of NDN on the improvement of pulmonary fibrosis through the regulation of an inflammatory response and the activation of the NLRP3 inflammasome using the PHMG-induced lung fibrosis model.

## 2. Results

### 2.1. Effects of NDN on Body Weight and Lung Weight

**Figure 1:**
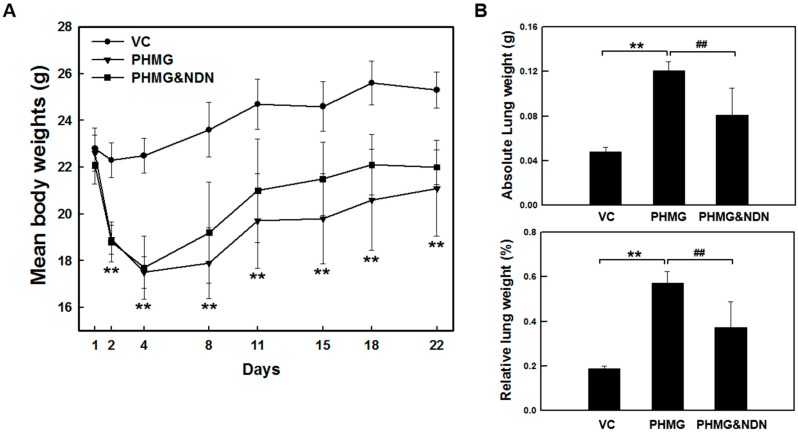
Effect of NDN on body and lung weights of PHMG-treated mice. Mice were intratracheally instilled with 1.1 mg/kg PHMG or saline on day 1. The PHMG & NDN group received an oral administration of NDN on days 1 and 21. The body weights were measured on the indicated days (**A**). The absolute lung weight was measured on day 22, and the relative lung weight was calculated as the ratio of the left lung weight to body weight of each mouse (**B**). Data are expressed as means ± SD (*n* = 6/group). ** *p* < 0.01 vs. VC group, ## *p* < 0.01 vs. PHMG group.

A single intratracheal instillation of 1.1 mg/kg PHMG resulted in a significant decrease in body weight from day 2 to day 4. From day 4, mice in the PHMG group and PHMG & NDN (60 mg/kg) group gradually gained body weight. There was no significant difference in body weight between the PHMG group and the PHMG & NDN group (Figure 1A). However, PHMG group and PHMG & NDN group significantly affected absolute and relative lung weights (Figure 1B). The PHMG treatment caused a significant increase in the absolute and relative lung weights when compared with that in the vehicle control (VC) group. Compared to the PHMG group, both the absolute and relative lung weights significantly decreased in the PHMG & NDN group.

### 2.2. Effects of NDN on Total and Differential Cell Counts in Bronchoalveolar Lavage Fluid (BALF)

**Figure 2:**
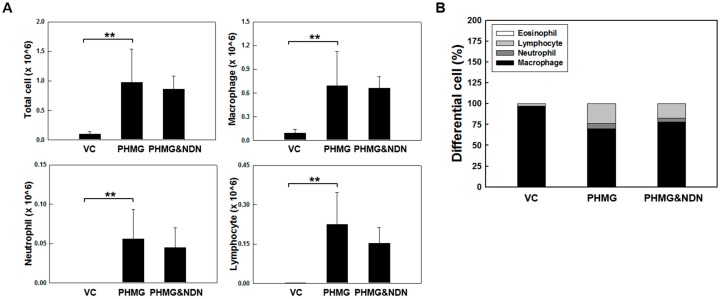
Effect of NDN on PHMG-induced changes in total and differential cell counts in the BALF of mice. The number of total cells, macrophages, neutrophils, and lymphocytes (**A**) in the BALF and composition of cell populations as a percentage of total cells (**B**) in VC, PHMG, and PHMG & NDN groups. Data are presented as the means ± SD (*n* = 6/group). ** *p* < 0.01 vs. VC group.

To evaluate the effects of NDN on the inflammatory response induced by PHMG, we measured cell counts of the BALF. The PHMG treatment induced extensive infiltration of inflammatory cells, as demonstrated by a significant increase in the number of total cells, macrophages, neutrophils, and lymphocytes in the PHMG group compared with those of the VC group. Although not significant, differences were observed between the groups, where the number of total cells, neutrophils, and lymphocytes decreased to approximately 11.2%, 19.7%, and 32%, respectively, in the PHMG & NDN group compared to those of the PHMG group (Figure 2A). Additionally, the ratio of neutrophil and lymphocyte to the total cell number increased in the PHMG group, while the ratio of macrophage to total cell number decreased in the PHMG group compared to the VC group (Figure 2B). The ratio of neutrophil and lymphocyte to total cell number decreased in the PHMG & NDN group, while the ratio of macrophage to total cell number increased in the PHMG & NDN group compared to the PHMG group (Figure 2B). However, no significant changes were observed in the number of eosinophils in both PHMG and PHMG & NDN groups.

### 2.3. Effects of NDN on Lung Histopathological Analysis and Hydroxyproline Content (HC)

**Figure 3:**
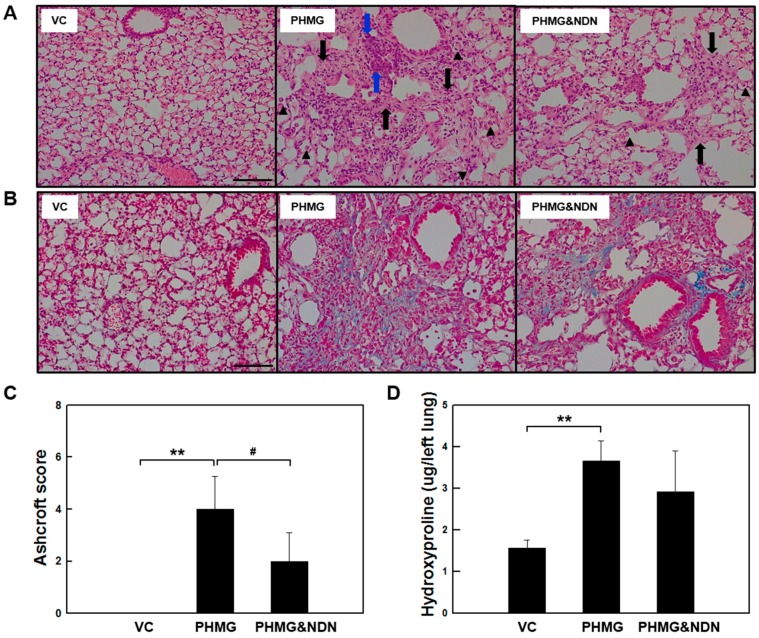
Effect of NDN on histopathological analysis and HC of the lung tissues. Representative histological sections from lung tissues. The lung sections were stained with H&E (**A**) and MT (**B**). The lung sections from the VC group showed a normal architecture. Black arrows indicate granulomatous inflammation/fibrosis, blue arrows indicate bronchioloalveolar epithelial hyperplasia (BEH), and black triangles indicate infiltration of alveolar macrophage. Scale bar represents 100 μm; (**C**) Ashcroft score was compared among the experimental groups. (**D**) Collagen content in the homogenates of the lung tissues was measured using a hydroxyproline assay (*n* = 6/group). Results are expressed as the means ± SD. ** *p* < 0.01 vs. VC group, # *p* < 0.05 vs. PHMG group.

In histologic analysis, lung fibrosis was assessed using the Ashcroft method as a semi-quantitative scoring system. The degree of lung fibrosis was graded on a scale (from 0 to 8), using the average of microscopic field scores. Lung HC was used as an indirect marker of collagen deposition. Histopathological analysis and HC changes identified in the lungs are presented in Figure 3 and Table 1. The VC group presented the left lung, which has a normal histological appearance (Figure 3A and Table 1). However, the lung in the PHMG group showed extensive damage characterized by granulomatous inflammation/fibrosis, bronchioloalveolar epithelial hyperplasia (BEH), and infiltration of the alveolar macrophages. These histological changes were less extensive in the PHMG & NDN group (Figure 3A and Table 1). The Ashcroft score was significantly increased in the PHMG group. However, the increase was significantly reduced in the PHMG & NDN group (Figure 3C). As assessed with Masson’s trichrome (MT) staining, the PHMG group exhibited severe fibrotic lesions and collagen deposition compared to the VC group (Figure 3B). These histopathological changes were improved in the PHMG & NDN group (Figure 3A,B and Table 1). The lung HC level, an index of collagen deposition, was increased approximately two-fold in the PHMG group compared to that in the VC group (Figure 3D), while the HC level decreased in the PHMG & NDN group compared to that of the PHMG group.

### 2.4. Effects of NDN on Inflammatory Cytokines Expression

**Figure 4:**
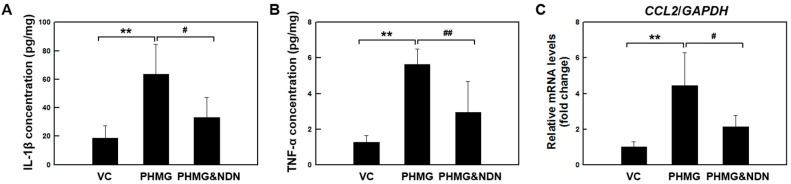
Effect of NDN on the expression of inflammatory factors of mice. The production of IL-1β and TNF-α in the lung tissue was detected by ELISA (**A**,**B**). The expression level of CCL2 was determined using qRT-PCR (**C**). Gene expression was presented as fold changes and normalized to GAPDH. Data are presented as the means ± SD (*n* = 6/group). ** *p* < 0.01 vs. VC group, # *p* < 0.05 and ## *p* < 0.01 vs. PHMG group.

Our recent study observed that intratracheal instillation of PHMG in the lungs of mice induced the expression of inflammatory cytokines such as interleukin 1 beta (IL-1β), tumor necrosis factor alpha (TNF-α), and CC chemokine ligand 2 (CCL2) [19,20]. To investigate whether NDN modulates the production of pro-inflammatory cytokines, the levels of IL-1β and TNF-α in lung tissues were evaluated using ELISA. The levels of IL-1β and TNF-α significantly increased in the PHMG group compared to those in the VC group, and IL-1β and TNF-α were markedly decreased in the PHMG & NDN group (Figure 4A–C). The mRNA expression of CCL2 significantly increased in the PHMG group compared to the VC group. However, the increase was significantly reduced in the PHMG & NDN group compared to that of the PHMG group (Figure 4C), and these expressions are illustrated in Figure 4C.

### 2.5. Effects of NDN on Fibrosis Relative Factors Expression in the Lungs

**Figure 5:**
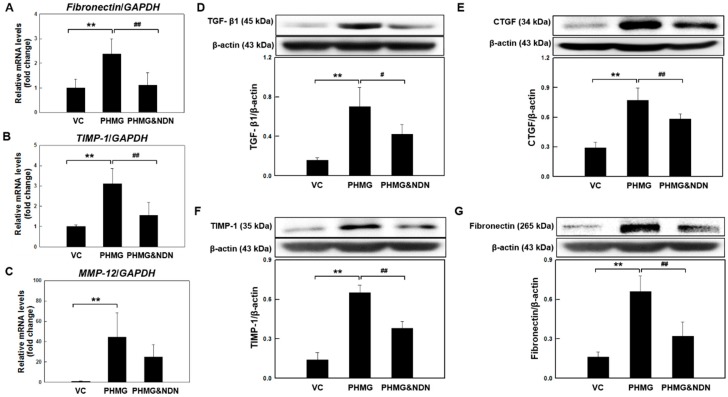
Effect of NDN on fibrotic factors expression of mice. The expression levels of fibronectin, TIMP-1, and MMP-12 mRNA were determined using qRT-PCR (**A**–**C**). The levels of CTGF, TGF- β1, TIMP-1, and fibronectin expressions in the lung tissues were determined by western blotting (**D**–**G**). Gene expressions were presented as fold changes and normalized to GAPDH. The blots were analyzed by densitometry and normalized to β-actin. Data are expressed as means ± SD (*n* = 6/group). ** *p* < 0.01 vs. VC group, # *p* < 0.05 and ## *p* < 0.01 vs. PHMG group.

We next examined the effects of NDN on the expression of fibrosis-related factors. Expression of fibrosis-related factors such as transforming growth factor beta (TGF-β1), matrix metalloproteinase 12 (MMP-12), tissue inhibitor of metalloproteinases-1 (TIMP-1), connective tissue growth factor (CTGF), and fibronectin, have been associated with the development of lung fibrosis. The mRNA expression of fibronectin, TIMP-1, and MMP-12 is shown in Figure 5A–C. Fibronectin, TIMP-1, and MMP-12 mRNA levels significantly increased in the PHMG group compared to the levels in the VC group. However, the increases were attenuated in the PHMG & NDN group. The protein expression of CTGF, TGF-β1, TIMP-1, and fibronectin is shown in Figure 5D–G. The protein expression levels of CTGF and TGF-β1, TIMP-1, and fibronectin significantly increased in the PHMG group. However, the increases were significantly reduced in the PHMG & NDN group (Figure 5D–G).

### 2.6. Effects of NDN on NLRP3 Inflammasome Activation in the Lungs

**Figure 6:**
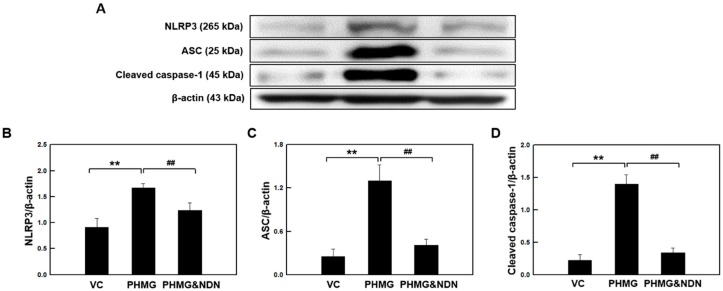
Effect of NDN on the expression of the NLRP3 inflammasome of mice. The levels of NLRP3, ASC, and cleaved caspase-1 expressions in the lung tissues were evaluated by western blotting (**A**–**D**). The blots were analyzed by densitometry and normalized to β-actin. Data are presented as the means ± SD (*n* = 6/group). ** *p* < 0.01 vs. VC group, ## *p* < 0.01 vs. PHMG group.

Our data demonstrated that NDN inhibited the secretion of IL-1β in the lung tissues of the PHMG group (Figure 4A). NLRP3 inflammasome activation drives the secretion of mature IL-1β alongside the caspase-1 cleavage-dependent pathway. The NLRP3 inflammasome is composed of NLRP3, an apoptosis-associated speck-like protein containing a caspase recruitment domain (ASC), and cysteine protease caspase-1 [22,23]. PHMG induced an increase in the protein levels of NLRP3 and ASC, while cleaved caspase-1 was dramatically decreased in the PHMG & NDN group (Figure 6A–D).

## 3. Discussion

NDN is a next generation, potent, small molecule TKs inhibitor that targets multiple TKs. NDN was approved by the FDA and has been developed for the treatment of a number of cancer types. Previous studies reported that NDN not only exhibits effective antitumor activity, but also beneficial therapeutic effects in pulmonary fibrosis, and is currently used in the treatment of IPF [24]. The clinical trials of NDN showed a significant reduction of forced vital capacity (FVC) in IPF patients, demonstrating that NDN should become the standard treatment for IPF patients [14,25]. Many people are troubled with pulmonary diseases due to PHMG inhalation. NDN is also used for the treatment of patients with pulmonary fibrosis by PHMG, but its mechanism of action in improving the disease has not been studied yet. Wollin et al. (2015) reported that NDN inhibits inflammatory responses, thus reducing the development of lung fibrosis in bleomycin (BLM)- and silica-induced lung fibrosis models [26]. In this study, we investigated the effects of NDN on the mitigation of pulmonary fibrosis through the reduction of an inflammatory response in the PHMG-induced lung fibrosis model. The results of this study showed that NDN inhibits the inflammatory response and the NLRP3 inflammasome activation of lung fibrosis induced by PHMG.

Our data demonstrated that intratracheal instillation of PHMG results in a significant decrease in body weight and a significant increase in lung weights. The changes in body and lung weights in the PHMG & NDN group were considerably alleviated compared to those in the PHMG group (Figure 1A,B). Lung injury is followed by an increase in the influx of inflammatory cells [27]. The presence of inflammatory cells in the BALF may indirectly reflect the degree of lung inflammation. Previous studies on pulmonary fibrosis by a single instillation of PHMG have shown that the number of total cells, macrophages, neutrophils, and lymphocytes increased in the BALF of mice [17,19]. The infiltration of neutrophils in the lungs is an early step in the pulmonary inflammatory process that leads to lung injury [28]. Previous studies have shown the infiltration of neutrophils up to four weeks in BALF with PHMG treatment. Our previous study showed the continuous infiltration of neutrophils in the BALF of PHMG-treated mice for up to four weeks [19]. Additionally, most of the inflammatory cells induced by PHMG were macrophages and lymphocytes, which mainly induce chronic inflammation [19,29,30]. Our BALF results are consistent with these findings in the PHMG group (Figure 2A,B). A previous study reported that NDN significantly reduced neutrophils or lymphocytes but not macrophages in the BALF of the BLM- and silica-induced lung fibrosis model [31]. These results are consistent with the data of our study (Figure 2A,B).

The PHMG caused major histopathological changes evidenced by the alveolar macrophages infiltration, BEH, and granulomatous inflammation/fibrosis in the lungs of mice [17,19,21]. Wollin et al. (2014) reported that NDN reduced lung inflammation, granuloma formation, and fibrosis in BLM- and silica-induced pulmonary fibrosis in mice [31]. Our results also showed that PHMG-induced histopathological changes were markedly alleviated by NDN treatment (Figure 3A,B, Table 1). Moreover, the Ashcroft scoring and HC were consistent with our histopathological findings (Figure 3C,D). These results suggest that NDN may be effective against PHMG-induced lung injury.

We also tested the effect of NDN on the expression of inflammatory cytokines associated with pulmonary injury. Inflammatory cytokines are released mainly from inflammatory cells in the lungs, and are considered to play a crucial role in the initiation and progression of pulmonary fibrosis [19,20,21]. Our previous studies showed that PHMG increases the expression levels of various inflammatory cytokines in the lungs of mice [19,20,21]. In the present study, PHMG induced a significant increase in the expression levels of inflammatory cytokines such as IL-1β, TNF-α, and CCL2. The expression of these cytokines in the lungs of mice in the PHMG & NDN group was significantly decreased compared to that in the PHMG group (Figure 4A–C). NDN has been shown to exhibit anti-inflammatory effects in the acute liver fibrogenesis mouse model [32]. Additionally, NDN exerted anti-inflammatory activity, demonstrated by diminished inflammatory cytokines in the lung fibrosis model [31]. Kong et al. (1997) reported that inhibitory activity on TKs reduced the expression of inflammatory cytokines including IL-6 and IL-1 [33]. The anti-inflammatory effects of NDN also might be partly attributable to the inhibitory activity on these TKs [34]. These results suggest that the anti-inflammatory effects of NDN may be involved in the alleviation of PHMG-induced lung injury.

We next examined the effect of NDN on the expression of fibrosis-related factors. Increased expression of TGF-β1, MMP-12, TIMP-1, CTGF, and fibronectin have been associated with the development of pulmonary fibrosis [35,36,37,38]. Autocrine signaling of IL-18 or IL-1β mediated by the activation of the NLRP3 inflammasome could promote the expression of TGF-β1 [39]. The TGF-β1, a major inducer of epithelial mesenchymal transition, induces the up-regulation of mesenchymal relative growth factors and extracellular matrix (ECM) proteins [37,38]. Wollin et al. (2014) reported that NDN reduced the expression of TGF-β1 in BLM-induced pulmonary fibrosis in rats [31]. Our data showed that the expression level of TGF-β1 in the lungs of the PHMG & NDN group was significantly decreased compared with that in the PHMG group (Figure 5E). The fibrotic response of TGF-β1 is also partly mediated by the production of several growth factors, including platelet-derived growth factor (PDGF), fibroblast growth factor 2 (FGF-2), and CTGF [40,41,42,43,44,45]. Previous studies reported that fibrogenic growth factors induce fibroblast focus formation and the excessive deposition of ECM, leading to tissue fibrosis [46].

Additionally, the imbalance of MMPs and TIMPs has been observed in several chronic pulmonary diseases such as IPF, asthma, and COPD [47,48,49,50]. PHMG causes the excessive production of ECM and modification of the expression of MMPs and TIMPs [43]. Our data showed that PHMG induced a significant increase in the expression of TIMP-1, MMP-12, CTGF, and fibronectin, and these factors in the lungs of mice in the PHMG & NDN group were significantly decreased compared with those in the PHMG group (Figure 5A–D,F,G). Meanwhile, the modulation of growth factors is regarded as a new therapeutic strategy for the treatment of fibrosis. NDN is also known to be a potent inhibitor of proangiogenic pathway receptor families such as FGF, vascular endothelial growth factor (VEGF), and PDGF receptors, indicating indirect inhibitory effects on TGF-β signaling [26]. These results suggest that NDN could markedly inhibit the expression of PHMG-induced fibrosis-related factors.

IL-1β can drive the progression of pulmonary fibrosis [51], and it is known to mediate the release of other inflammatory cytokines such as TNF-α and IL-6 [52]. Inactive pro-IL-1β and pro-IL-18 are cleaved by caspase-1, and maturation of caspase-1 is promoted by NLRP3 inflammasome activation [53,54]. The NLRP3 inflammasome is known to play an important role in inflammation and fibrosis [55]. Caspase-1 and NLRP3 levels were shown to increase in IPF patients [56]. A previous study showed that the activation of the NLRP3 inflammasome was observed in BLM-induced lung fibrosis, and NLPR3 deficiency mitigates lung fibrosis [57]. In addition, BLM-induced lung collagen production was mitigated in mice deficient in either caspase-1 or NLRP3, and inflammatory and pro-fibrotic mediators were significantly reduced in NLRP3- and ASC-deficient mice [55]. Our previous study demonstrated that PHMG induced the activation of the NLRP3 inflammasome [17], and similar findings were obtained in the present study. However, we found that activation of the NLRP3 inflammasome was markedly attenuated by NDN treatment (Figure 4D–G). These results suggest that the inhibition of NLRP3 inflammasome activation by NDN may at least partially reduce the inflammatory response in PHMG-induced lung fibrosis.

## 4. Materials and Methods

### 4.1. Animals and Environmental Conditions

Male C57BL/6 mice (aged seven weeks) were purchased from Orient Bio Inc. (Seongnam, Korea) and used after one week of quarantine and acclimation. The mice were housed in a room maintained at a temperature of 22 ± 3 °C, relative humidity of 50 ± 20%, light intensity of 150–300 lux, light/dark cycle of 12/12 h, and air ventilation refreshed 10–20 times/h. The mice were fed pelleted food for experimental animals (PMI Nutrition International, Richmond, IN, USA) and UV-irradiated (Steritron SX-1; Daeyoung Inc., Seoul, Korea) and filtered (1 μm) tap water was provided ad libitum. The experimental procedures were approved by the Institutional Animal Care and Use Committee of the Korea Institute of Toxicology (IACUC #1708-0306).

### 4.2. Test Chemicals and Treatment

The PHMG solution (25%) we used was a generous gift from SK Chemicals (Seongnam, Korea). NDN (i.e., BIBF 1120) was purchased from Selleck Chemicals Co. Ltd. (Houston, TX, USA) with purity > 99%. Mice in the PHMG group received a single intratracheal instillation of 1.1 mg/kg, in 50 μL saline solution using an automatic video instillator [58]. Mice in the VC group were instilled with saline through the same route. NDN was suspended in a 0.5% sodium carboxymethylcellulose (CMC, Sigma-Aldrich, Saint Louis, MO, USA) solution and was gavaged to mice once daily for a period of 21 days at 60 mg/kg/day. The daily application volume of NDN was calculated in advance based on the most recently recorded body weight of the individual animal. Mice in the VC and PHMG groups received 0.5% CMC instead of NDN via the same route.

### 4.3. Experimental Design and Dose Selection

Mice were assigned randomly to the following experimental groups: (1) VC; (2) PHMG (1.1 mg/kg); and (3) PHMG & NDN (60 mg/kg) group (*n* = 6/group). The dose of PHMG used in the present study was selected based on our previous studies of induced lung injury with moderate granulomatous inflammation/fibrosis in mice [17,19,20,21]. The effective NDN dose was based on earlier studies [28]. There was no study of NDN treatment using a single therapy in the fibrosis model. Body weights were measured prior to the first instillation, and on days 2, 4, 8, 11, 15, and 18. On the scheduled termination day (day 22 of the study), the terminal body weight of each mouse was measured, after which they were euthanized by exsanguination. Clinical signs of mice in each group were monitored every day. Bronchoalveolar lavage was performed for the right lung lobes and the lungs were removed. The left lung lobe was weighed and fixed in 10% neutral-buffered formalin (NRF). The right lung lobes were snap-frozen and stored at −80 °C until analysis.

### 4.4. BALF Preparation

BALF was performed in the right lung through a tracheal cannula during exsanguination using 0.7 mL phosphate buffered saline (PBS) three times in each mouse. Total cell counts in the BALF were performed using a disposable hemocytometer (INCYTO, Seoul, Korea). A 0.2 mL aliquot of the cell suspension was centrifuged (Shandon Cytospin 4; Thermo, Waltham, MA, USA) at 800 rpm for 10 min onto a glass slide. The slides were stained with Diff-Quik stain (Sysmex, Kobe, Japan), and then washed, air-dried, and observed by a light microscope (BX51; Olympus, Tokyo, Japan). At least 200 cells included macrophages, neutrophils, and lymphocytes per sample were scored.

### 4.5. Histopathological Examination

The left lung tissue was fixed in 10% NRF over 24 h, embedded in paraffin, and sectioned to 4-µm thick slices. Lung sections were stained with hematoxylin and eosin (H&E; Sigma-Aldrich, Saint Louis, MO, USA) for histological analysis and MT (Sigma-Aldrich, Saint Louis, MO, USA) for examination of fibrotic changes. Histopathological analysis of the lung tissue sections was performed using a light microscope at 200× magnification. Histopathological changes were scored on a scale ranging from 0 to 5. Each successive field was individually assessed to determine the severity of BEH, granulomatous inflammation/fibrosis, and macrophage infiltration in the alveoli. A fibrosis score was assigned ranging from 0 to 8 using the Ashcroft scoring system for each animal [59].

### 4.6. Lung Hydroxyproline Assay

The collagen content of the right lung tissues was determined using a hydroxyproline colorimetric assay kit (Biovision Inc., Milpitas, CA, USA) according to the manufacturer’s protocol. Following this, 0.1 mL of homogenized lung sample was quickly transferred to a cryovial tube (SPL, Gyeonggi-Do, Korea). Another 0.1 mL of 12 M hydrochloric acid (HCl, Sigma-Aldrich, Zwijndrecht, The Netherlands) was added, and hydrolyzed at 120 °C for 3 h. The sample was centrifuged at 10,000× *g* for 3 min at room temperature (RT), and the supernatant was diluted two-fold with distilled water. The 10 μL of diluted supernatant sample was transferred to a 96-well plate and evaporated to dryness at 60 °C; a standard curve performed according to the manufacturer’s protocol. Subsequently, 0.1 mL of chloramine T reagent/oxidation buffer mixture was added to each well and incubated for 5 min at RT. Finally, 0.1 mL of para-dimethylaminobenzaldehyde reagent was added to each well and incubated for 90 min at 60 °C. A Model 680 Plate Reader (Bio-Rad Laboratories, Hercules, CA, USA) was used to measure the absorbance at 540 nm.

### 4.7. Western Blot Analysis

Frozen lung tissues were homogenized in ice-cold RIPA Buffer (Pierce Biotechnology, Rockford, IL, USA) with a protease inhibitor cocktail (Roche, Mannheim, Germany). The homogenates were then centrifuged at 13,400× *g* for 20 min at 4 °C to obtain the supernatants. The protein concentration of supernatants was measured using the bicinchoninic acid (BCA, Sigma-Aldrich, St. Louis, MO, USA) method. Protein lysates were separated by SDS-PAGE and then transferred onto polyvinylidene difluoride membranes (Millipore, Billerica, MA, USA). The membranes were blocked using 5% skimmed milk in Tris-buffered saline with Tween^®^ 20 (TBS-T) for 90 min at RT. Primary antibodies were diluted in 5% skimmed milk and incubated overnight at 4 °C with gentle shaking. The membranes were washed five times with TBS-T and incubated for 1 h at RT with horseradish peroxidase-conjugated secondary antibodies in TBS-T containing 5% skimmed milk. After five washes with TBS-T, the membranes were detected using chemiluminescence reagents (Pierce Biotechnology, Rockford, IL, USA) according to the manufacturer’s instructions. Anti-TGF-β1 was purchased from Abcam (Cambridge, UK), while anti-CTGF was purchased from Thermo Fisher Scientific (Cambridge, MA, USA). Anti-fibronectin, anti-NLRP3, anti-ASC, and anti-caspase-1 were purchased from Novus Biologicals (Littleton, CO, USA). Anti-β-actin and all secondary antibodies were purchased from Santa Cruz Biotechnology (Santa Cruz, CA, USA). The relative intensity of the bands was quantified using the ImageJ software, and all the results were normalized to β-actin.

### 4.8. Tissue Cytokine Analysis by ELISA

Mouse TNF-α was measured in the BALF by a commercial ELISA kit (R&D Systems, Minneapolis, MN, USA), according to the manufacturer’s protocol. The levels of IL-1β were determined in lung tissue using the Quantikine ELISA Kit (R&D Systems, Minneapolis, MN, USA), also according to the manufacturer’s protocol. The tissue homogenate protein levels were measured using the BCA method, and cytokine values were normalized to protein levels. All samples and standards were measured in duplicate.

### 4.9. Quantitative Real Time-PCR (qRT-PCR)

Total RNA from right lung tissues was isolated using the RNeasy Mini Kit (Qiagen, Valencia, CA, USA) according to the manufacturer’s protocol, and quantified using a NanoDrop 2000 spectrophotometer (Thermo Scientific, Wilmington, DE, USA). Reverse transcription was performed on 500 ng of total RNA using the First Strand cDNA Synthesis kit (Takara, Kyoto, Japan) according to the manufacturer’s instructions. Sequences of the mouse gene-specific primers were used as follows: fibronectin (forward: 5′-CACGATGCGGGTCACTTG-3′, reverse: 5′-CTGCAACGTCCTCTTCATTCTTC-3′), glyceraldehyde 3-phosphate dehydrogenase (GAPDH, forward: 5′-ATCACCATCTTCCAGGAGCGA-3′, reverse: 5′-AGGGGCCATCCACAGTCTT-3′), CCL2 (forward: 5′-TTGTCACCAAGCTCAAGAGAGA-3′, reverse: 5′-GAGGTGGTTGTGGAAAAGGTAG-3′), TIMP-1 (forward: 5′-ATTCAAGGCTGTGGGAAATG-3′, reverse: 5′-CTCAGAGTACGCCAGGGAAC-3′), and MMP-12 (forward: 5′-CACAACAGTGGGAGAGAAAA-3′, reverse: 5′-AGCTTGAATACCAGATGGGATG-3′). qRT-PCR was performed using the Power SYBR® Green Master Mix (Applied Biosystems, Foster City, CA, USA) with StepOnePlus™ Real-Time PCR Systems (Applied Biosystems). The level of gene expression of each transcript was normalized to the internal control gene (GAPDH). Relative gene expression was calculated using the ΔΔCt method, where Ct = threshold cycle.

### 4.10. Statistics

Statistical analyses were performed using GraphPad InStat v. 3.0 (GraphPad Software, Inc., La Jolla, CA, USA). Data are presented as mean ± standard deviation (SD) and all statistical comparisons were performed using one-way analysis of variance followed by a Student’s two-tailed *t*-test with *p*-values of *p* < 0.01 or *p* < 0.05 considered statistically significant.

## 5. Conclusions

In the present study, we evaluated the effects of NDN on PHMG-induced pulmonary injury in mice. Our results indicate that NDN reduced inflammatory events, including the inhibition of the expression of inflammatory cytokines and inflammatory cell infiltration. NDN also decreased the expression of fibrosis-related factors, and histopathological changes in the lungs of mice treated with PHMG were improved. Moreover, NDN significantly suppressed the activation of the NLRP3 inflammasome induced by PHMG, which is considered to play a key role in inflammation and fibrosis. These results suggest that NDN may modulate the inflammatory and fibrotic responses, thus reducing the development of PHMG-induced pulmonary fibrosis.

## Figures and Tables

**Table 1:** Quantitative histopathological assessment of the lung sections.

Group	VC	PHMG	PHMG & NDN
Number of animals	6	6	6
Hyperplasia, bronchiolo-alveolar	(0)	(4)	(1)
Minimal	0	0	1
Moderate	0	3	0
Severe	0	1	0
Mean ± SD	0	2.2 ± 1.7	0.2 ± 0.4 *
granulomatous inflammation/fibrosis	(0)	(6)	(6)
Slight	0	0	2
Moderate	0	1	2
Severe	0	5	2
Mean ± SD	0	3.8 ± 0.4	3.0 ± 0.9
Alveolar macrophage infiltration	(0)	(6)	(4)
Minimal	0	0	1
Slight	0	3	2
Moderate	0	3	1
Mean ± SD	0	2.5 ± 0.5	1.3 ± 1.2

0: no symptoms; 1: minimal; 2: slight; 3: moderate; 4: marked; 5: severe. Data are presented as mean ± SD (*n* = 6/group). PHMG group vs. PHMG & NDN group: * *p* < 0.05.

## References

[1] Burnham E.L., Janssen W.J., Riches D.W. (2014). The fibroproliferative response in acute respiratory distress syndrome: Mechanisms and clinical significance. Eur. Respir. J..

[2] Cabrera-Benitez N.E., Laffey J.G., Parotto M. (2014). Mechanical ventilation-associated lung fibrosis in acute respiratory distress syndrome a significant contributor to poor outcome. Anesthesiology.

[3] Raghu G., Collard H.R., Egan J.J. (2011). An official ATS/ERS/JRS/ALAT statement: Idiopathic pulmonary fibrosis: Evidence-based guidelines for diagnosis and management. Am. J. Respir. Crit. Care Med..

[4] Gharaee-Kermani M., Hu B., Thannickal V.J., Phan S.M., Gyetko M.R. (2017). Current and emerging drugs for idiopathic pulmonary fibrosis. Expert Opin. Emerg. Drugs.

[5] Thannickal V.J., Flaherty K.R., Hyzyrcl R.C., Lynch J.P. (2005). Emerging drugs for idiopathic pulmonary fibrosis. Exp. Opin. Emerg. Drugs.

[6] Rafii R., Juarez M.M., Albertson T.E., Chan A.L. (2013). A review of current and novel therapies for idiopathic pulmonary fibrosis. J. Thorac. Dis..

[7] Aleshina E.Y., Yudanova T.N., Skokova I.F. (2001). Production and Properties of Polyvinyl Alcohol Spinning Solutions Containing Protease C and Polyhexamethylene Guanidine. Fibre Chem..

[8] Korea Centers for Disease Control and Prevention (2011). Interim report of epidemiological investigation on lung injury with unknown cause in Korea. Public Health Wkly. Rep. KCDC.

[9] Hong S.B., Kim H.J., Huh J.W., Do K.H., Jang S.J., Song J.S., Choi S.J., Heo Y., Kim Y.B., Lim C.M. (2014). Korean Unknown Severe Respiratory Failure Collaborative, the Korean Study Group of Respiratory Failure. A cluster of lung injury associated with home humidifier use: Clinical, radiological and pathological description of a new Syndrome. Thorax.

[10] Hilberg F., Roth G., Krssak M., Kautschitsch S., Sommergruber W., Tontsch-Grunt U., Garin-Chesa P., Bader G., Zoephel A., Quant J. (2008). BIBF 1120: Triple angiokinase inhibitor with sustained receptor blockade and good antitumor efficacy. Cancer Res..

[11] Kudo K., Arao T., Tanaka K., Nagai T., Furuta K., Sakai K., Kaneda H., Matsumoto K., Tamura D., Aomatsu K. (2011). Antitumor Activity of BIBF 1120, a Triple Angiokinase Inhibitor, and Use of VEGFR2+pTyr+ Peripheral Blood Leukocytes as a Pharmacodynamic Biomarker in Vivo. Clin. Cancer Res..

[12] Keating G.M. (2015). Nintedanib: A Review of Its Use in Patients with Idiopathic Pulmonary Fibrosis. Drugs.

[13] Fala L. (2015). Ofev (Nintedanib): First Tyrosine Kinase Inhibitor Approved for the Treatment of Patients with Idiopathic Pulmonary Fibrosis. Am. Health Drug Benefits.

[14] Richeldi L., du Bois R.M., Raghu G., Azuma A., Brown K.K., Costabel U., Cottin V., Flaherty K.R., Hansell D.M., Inoue Y. (2014). Efficacy and safety of nintedanib in idiopathic pulmonary fibrosis. N. Engl. J. Med..

[15] Allen I.C., Scull M.A., Moore C.B., Holl E.K., McElvania-TeKippe E., Taxman D.J., Guthrie E.H., Pickles R.J., Ting J.P. (2016). The NLRP3 Inflammasome Mediates In Vivo Innate Immunity to Influenza A Virus through Recognition of Viral RNA. Immunity.

[16] Wynn T.A. (2011). Integrating mechanisms of pulmonary fibrosis. J. Exp. Med..

[17] Kim M.S., Han J.Y., Kim S.H., Jeon D., Kim H.Y., Lee S.W., Rho M.C., Lee K. (2018). Oleanolic acid acetate attenuates polyhexamethylene guanidine phosphate-induced pulmonary inflammation and fibrosis in mice. Respir Physiol. Neurobiol..

[18] Saitoh T., Akira S. (2010). Regulation of innate immune responses by autophagy-related proteins. J. Cell Biol..

[19] Kim M.S., Kim S.H., Jeon D., Kim H.Y., Lee K. (2018). Changes in expression of cytokines in polyhexamethylene guanidine-induced lung fibrosis in mice: Comparison of bleomycin-induced lung fibrosis. Toxicology.

[20] Song J.A., Park H.J., Yang M.J., Jung K.J., Yang H.S., Song C.W., Lee K. (2014). Polyhexamethyleneguanidine phosphate induces severe lung inflammation, fibrosis, and thymic atrophy. Food Chem. Toxicol..

[21] Song J., Kim W., Kim Y.B., Kim B., Lee K. (2018). Time course of polyhexamethyleneguanidine phosphate-induced lung inflammation and fibrosis in mice. Toxicol. Appl. Pharmacol..

[22] Tschopp J., Schroder K. (2010). NLRP3 inflammasome activation: The convergence of multiple signalling pathways on ROS production?. Nat. Rev. Immunol..

[23] Martinon F., Burns K., Tschopp J. (2002). The inflammasome: A molecular platform triggering activation of inflammatory caspases and processing of proIL-β. Mol. Cell.

[24] Ogura T., Taniguchi H., Azuma A., Inoue Y., Kondoh Y., Hasegawa Y., Bando M., Abe S., Mochizuki Y., Chida K. (2015). Safety and pharmacokinetics of nintedanib and pirfenidone in idiopathic pulmonary fibrosis. Eur. Respir. J..

[25] Richeldi L., Costabel U., Selman M., Kim D.S., Hansell D.M., Nicholson A.G., Brown K.K., Flaherty K.R., Noble P.W., Raghu G. (2011). Efficacy of a tyrosine kinase inhibitor in idiopathic pulmonary fibrosis. N. Engl. J. Med..

[26] Wollin L., Wex E., Pautsch A., Schnapp G., Hostettler K.E., Stowasser S., Kolb M. (2015). Mode of action of nintedanib in the treatment of idiopathic pulmonary fibrosis. Eur. Respir. J..

[27] Janick-Buckner D., Ranges G.E., Hacker M.P. (1989). Alteration of bronchoalveolar lavage cell populations following bleomycin treatment in mice. Toxicol. Appl. Pharmacol..

[28] Abraham E. (2003). Neutrophils and acute lung injury. Crit. Care Med..

[29] Shaw R.J. (1991). The role of lung macrophages at the interface between chronic inflammation and fibrosis. Respir. Med..

[30] Vanaudenaerde B.M., Verleden S.E., Vos R., De Vleeschauwer S.I., Willems-Widyastuti A., Geenens R., Van Raemdonck D.E., Dupont L.J., Verbeken E.K., Meyts I. (2011). Innate and adaptive interleukin-17-producing lymphocytes in chronic inflammatory lung disorders. Am. J. Respir. Crit. Care Med..

[31] Wollin L., Maillet I., Quesniaux V., Holweg A., Ryffel B. (2014). Antifibrotic and Anti-inflammatory Activity of the Tyrosine Kinase Inhibitor, Nintedanib, in Experimental Models of Lung Fibrosis. J. Pharmacol. Exp. Ther..

[32] Öztürk Akcora B., Storm G., Prakash J., Bansal R. (2017). Tyrosine kinase inhibitor BIBF1120 ameliorates inflammation, angiogenesis and fibrosis in CCl4-induced liver fibrogenesis mouse model. Sci. Rep..

[33] Kong L.Y., Lai C., Wilson B.C., Simpson J.N., Hong J.S. (1997). Protein tyrosine kinase inhibitors decrease lipopolysaccharide-induced proinflammatory cytokine production in mixed glia, microglia-enriched or astrocyte-enriched cultures. Neurochem. Int..

[34] Das J., Chen P., Norris D., Padmanabha R., Lin J., Moquin R.V., Shen Z., Cook L.S., Doweyko A.M., Pitt S. (2006). 2-Aminothiazole as a novel kinase inhibitor template. Structure-activity relationship studies toward the discovery of *N*-(2-chloro-6-methylphenyl)-2-[[6-[4-(2-hydroxyethyl)-1-piperazinyl)]-2-methyl-4-pyrimidinyl]amino)]-1,3-thiazole-5-carboxamide (dasatinib, BMS-354825) as a potent pan-Src kinase inhibitor. J. Med. Chem..

[35] Manoury B., Caulet-Maugendre S., Guénon I., Lagente V., Boichot E. (2006). TIMP-1 is a key factor of fibrogenic response to bleomycin in mouse lung. Int. J. Immunopathol. Pharm..

[36] Garbacki N., Di Valentin E., Piette J., Cataldo D., Crahay C., Colige A. (2009). Matrix metalloproteinase 12 silencing: A therapeutic approach to treat pathological lung tissue remodeling?. Pulm. Pharmacol. Ther..

[37] Van Zijl F., Zulehner G., Petz M., Schneller D., Kornauth C., Hau M., Machat G., Grubinger M., Huber H., Mikulits W. (2009). Epithelial-mesenchymal transition in hepatocellular carcinoma. Future Oncol..

[38] Xu J., Lamouille S., Derynck R. (2009). TGF-beta-induced epithelial to mesenchymal transition. Cell Res..

[39] Artlett C.M. (2012). The Role of the NLRP3 Inflammasome in Fibrosis. Open Rheumatol. J..

[40] Chaudhary N.I., Roth G.J., Hilberg F., Mu¨ller-Quernheim J., Prasse A., Zissel G., Schnapp A., Park J.E. (2007). Inhibition of PDGF, VEGF and FGF signalling attenuates fibrosis. Eur. Respir. J..

[41] Li C.M., Khosla J., Pagan I., Hoyle P., Sannes P.L. (2000). TGF-β1 and fibroblast growth factor-1 modify fibroblast growth factor-2 production in type II cells. Am. J. Physiol. Lung Cell. Mol. Physiol..

[42] Kucich U., Rosenbloom J.C., Herrick D.J., Abrams W.R., Hamilton A.D., Sebti S.M., Rosenbloom J. (2001). Signaling events required for transforming growth factor-beta stimulation of connective tissue growth factor expression by cultured human lung fibroblasts. Arch. Biochem. Biophys..

[43] Kim H.R., Lee K., Park C.W., Song J.A., Shin D.Y., Park J.Y., Chung K.H. (2016). Polyhexamethylene guanidine phosphate aerosol particles induce pulmonary inflammatory and fibrotic responses. Arch. Toxicol..

[44] Roberts C.J., Birkenmeier T.M., McQuillan J.J., Akiyama S.K., Yamada S.S., Chen W.T., Yamada K.M., McDonald J.A. (1998). Transforming growth factor beta stimulates the expression of fibronectin and of both subunits of the human fibronectin receptor by cultured human lung fibroblasts. J. Biol. Chem..

[45] Ignotz R.A., Massague J. (1986). Transforming growth factor-beta stimulates the expression of fibronectin and collagen and their incorporation into the extracellular matrix. J. Biol. Chem..

[46] Inomata M., Nishioka Y., Azuma A. (2015). Nintedanib: Evidence for its therapeutic potential in idiopathic pulmonary fibrosis. Core Evid..

[47] Belvisi M.G., Bottomley K.M. (2003). The role of matrix metalloproteinases (MMPs) in the pathophysiology of chronic obstructive pulmonary disease (COPD): A therapeutic role for inhibitors of MMPs?. Inflamm. Res..

[48] Vignola A.M., Riccobono L., Mirabella A., Profita M., Chanez P., Bellia V., Mautino G., D’accardi R., Bousquet J., Bonsignore G. (1998). Sputum metalloproteinase-9/tissue inhibitor of metalloproteinase-1 ratio correlates with airflow obstruction in asthma and chronic bronchitis. Am. J. Respir. Crit. Care Med..

[49] Pardo A., Selman M., Ramirez R., Ramos C., Montaño M., Stricklin G., Raghu G. (1992). Production of collagenase and tissue inhibitor of metalloproteinases by fibroblasts derived from normal and fibrotic human lung. Chest.

[50] Murphy G., Docherty A.L. (1992). The matrix metalloproteinases and their inhibitors. Am. J. Respir. Cell Mol. Biol..

[51] Kolb M., Marqetts P.J., Anthony K.C., Pitossi F., Gauldie J. (2001). Transient expression of IL-1beta induces acute lung injury and chronic repair leading to pulmonary fibrosis. J. Clin. Investig..

[52] Borthwick L.A., Wynn T.A., Fisher A.J. (2013). Cytokine mediated tissue fibrosis. Biochem. Biophys. Acta.

[53] Strowig T., Henao Mejia J., Elinav E., Flavell R. (2012). Inflammasomes in health and disease. Nature.

[54] Cassel S.L., Joly S., Sutterwala F.S. (2009). The NLRP3 inflammasome: A sensor of immune danger signals. Semin. Immunol..

[55] Gasse P., Riteau N., Charron S., Girre S., Fick L., Petrilli V., Tschopp J., Lagente V., Quesniaux V.F., Ryffel B. (2009). Uric acid is a danger signal activating NALP3 inflammasome in lung injury inflammation and fibrosis. Am. J. Respir. Crit. Care Med..

[56] Lasithiotaki I., Giannarakis I., Tsitoura E., Samara K.D., Margaritopoulos G.A., Choulaki C., Vasarmidi E., Tzanakis N., Voloudaki A., Sidiropoulos P. (2016). NLRP3 inflammasome expression in idiopathic pulmonary fibrosis and rheumatoid lung. Eur. Respir. J..

[57] Stout-Delgado H.W., Cho S.J., Chu S.G., Mitzel D.N., Villalba J., El-Chemaly S., Ryter S.W., Choi A.M., Rosas I.O. (2016). Age-dependent susceptibility to pulmonary fibrosis is associated with NLRP3 inflammasome activation. Am. J. Respir. Cell Mol. Biol..

[58] Kim S.N., Lee J., Yang H.S., Cho S., Kim Y.B., Her J.D., Cho K.H., Song C.W., Lee K. (2010). Dose-response Effects of Bleomycin on Inflammation and Pulmonary Fibrosis in Mice. Toxicol. Res..

[59] Ashcroft T., Simpson J.M., Timbrell V. (1988). Simple method of estimating severity of pulmonary fibrosis on a numerical scale. J. Clin. Pathol..

